# Mobilization of Intracellular Copper by Gossypol and Apogossypolone Leads to Reactive Oxygen Species-Mediated Cell Death: Putative Anticancer Mechanism

**DOI:** 10.3390/ijms17060973

**Published:** 2016-06-20

**Authors:** Haseeb Zubair, Shafquat Azim, Husain Yar Khan, Mohammad Fahad Ullah, Daocheng Wu, Ajay Pratap Singh, Sheikh Mumtaz Hadi, Aamir Ahmad

**Affiliations:** 1 Department of Biochemistry, Faculty of Life Sciences, AMU, Aligarh 202002, India; hzubair@health.southalabama.edu (H.Z.); SAzim@health.southalabama.edu (S.A.); husainyar@gmail.com (H.Y.K.); m.ullah@ut.edu.sa (M.F.U.); 2 Department of Oncologic Sciences, Mitchell Cancer Institute, University of South Alabama, Mobile, AL 36604, USA; asingh@health.southalabama.edu; 3 UoN Chair of Oman’s Medicinal Plants and Marine Natural Products, University of Nizwa, Birkat Al Mauz, PO Box 33, 616 Nizwa, Oman; 4 Prince Fahad Research Chair, Department of Medical Laboratory Technology, Faculty of Applied Medical Sciences, University of Tabuk, P.O. Box 741, Tabuk-71491, Saudi Arabia; 5 Key Laboratory of Biomedical Information Engineering of Education Ministry, School of Life Science and Technology, Xi’an Jiaotong University, Xi’an 710048, China; wudaocheng@mail.xjtu.edu.cn

**Keywords:** gossypol, apogossypolone (ApoG2), copper, prooxidant, anticancer

## Abstract

There is compelling evidence that serum, tissue and intracellular levels of copper are elevated in all types of cancer. Copper has been suggested as an important co-factor for angiogenesis. It is also a major metal ion present inside the nucleus, bound to DNA bases, particularly guanine. We have earlier proposed that the interaction of phenolic-antioxidants with intracellular copper leads to the generation of reactive oxygen species (ROS) that ultimately serve as DNA cleaving agents. To further validate our hypothesis we show here that the antioxidant gossypol and its semi-synthetic derivative apogossypolone induce copper-mediated apoptosis in breast MDA-MB-231, prostate PC3 and pancreatic BxPC-3 cancer cells, through the generation of ROS. MCF10A breast epithelial cells refractory to the cytotoxic property of these compounds become sensitized to treatment against gossypol, as well as apogossypolone, when pre-incubated with copper. Our present results confirm our earlier findings and strengthen our hypothesis that plant-derived antioxidants mobilize intracellular copper instigating ROS-mediated cellular DNA breakage. As cancer cells exist under significant oxidative stress, this increase in ROS-stress to cytotoxic levels could be a successful anticancer approach.

## 1. Introduction

Cancer still remains a leading cause of deaths worldwide [1]. The development and progression of cancer is a dynamic and long-term process triggered by alterations in genetic sequences and acquiring of specific characteristics that enable development of full malignancy [2]. It has been suggested that even within a single type of tumor, different genomic signatures can be present in different transformed cells [3]. Moreover, a similar tumor in different individuals also tends to have different mutations and genetic structures involved. Based on the genomic and transcriptomic architecture of 2000 breast tumors, Curtis *et al.* [4] identified 10 novel subgroups of breast cancer. Despite the complexity of the carcinogenesis process and the different types of mutations, the arising characteristic changes are often a small number of molecular, biochemical, and cellular traits, which often lead to changes in the metabolic status of the tumor as compared to normal cells [5,6,7,8]. Thus, targeting the arising metabolic changes, unique to all types of cancer, rather than the mutations responsible for these metabolic changes can facilitate the development of potential anticancer agents at a faster rate.

We have previously shown that different classes of plant-derived antioxidants, such as polyphenols, induce oxidative breakage of cellular DNA alone or in presence of copper ions [9,10,11,12,13]. Observations made over decades have provided compelling evidence indicating significant elevation of serum, plasma and intracellular copper levels in all types of malignancies [14,15,16,17]. Copper is required by the tumor cells for the secretion of angiogenic factors, and stimulates proliferation and migration of endothelial cells [18]. While iron is considerably more abundant in normal biological systems, the major ions in the nucleus are copper and zinc [19,20]. In view of our findings and those of others in literature we suggest that the antioxidants possessing anticancer and apoptosis inducing activities mobilize copper ions, possibly endogenous chromatin-bound copper, and lead to the generation of reactive oxygen species [19,20]. Gossypol, a polyphenolic aldehyde produced in the roots, stem, and seeds of the cotton plant, has been shown to have antiproliferative property against a wide range of cancers (breast [21]; bladder [22]; pancreas [23]; lung [24]; colon [25]; prostate [26]; and head and neck [27,28]). Although gossypol is an effective antitumor compound *in vitro*, the toxicity of gossypol does not permit it to be used clinically. Many derivatives using gossypol as the parent compound have been synthesized [29,30].

Apogossypolone (ApoG2), a semi-synthetic analog of gossypol has shown promising results as an antitumor agent. However, the primary mechanism of action of these molecules is not known and has been the subject of considerable interest. We have previously shown that both gossypol and its derivative, ApoG2, cause oxidative DNA breakage in cells [31]. Furthermore, the greater permeability of ApoG2 results in the enhanced DNA breakage activity of the compound. In order to further establish our hypothesis, in this paper, using breast, prostate and pancreatic cancer cell lines, we further identify copper as a molecular target of these antioxidants. We show that the cell proliferation inhibition and apoptosis induction by gossypol and ApoG2 is mediated through the redox-cycling of copper and subsequent generation of reactive oxygen species (ROS).

## 2. Results

### 2.1. Gossypol and ApoG2 Specifically Inhibit Proliferation and Induce Apoptosis in Cancer Cells

**Figure 1:**
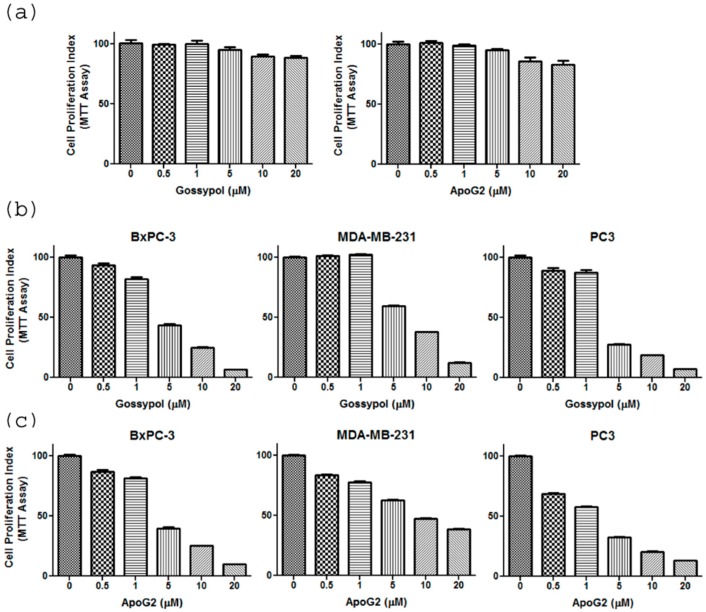
(**a**) The cell proliferation of normal breast epithelial cells (MCF10A) in presence of gossypol and ApoG2 was detected using MTT (3-(4,5-dimethylthiazol-2-yl)-2,5-diphenyltetrazolium bromide) assay. MCF10A cell were incubated with increasing concentration of gossypol/ApoG2, as indicated, for 72 h. Similarly, the cell proliferation of pancreatic cancer cell line, BxPC3; breast cancer cell line, MDA-MB-231; and prostate cancer cell line, PC3; in presence of increasing concentrations of (**b**) gossypol and (**c**) apogossypolone, as indicated, was detected using MTT assay. The cells were incubated for 72 h. MTT assay was performed as described in the Materials and Methods. All results are present as percentage of control (±SEM) of three independent experiments.

**Figure 2:**
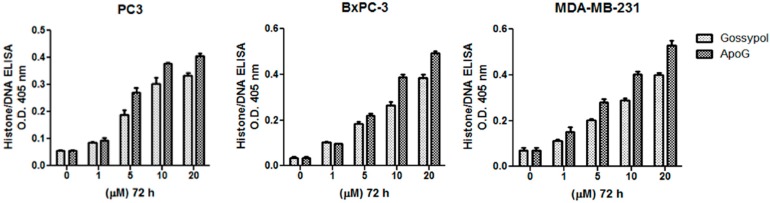
The Cell Death Detection ELISA Kit (Roche, Palo Alto, CA, USA) was used to detect apoptosis in MDA-MB-231, BxPC3 and PC3 cancer cell lines. Cells were treated with increasing concentrations of gossypol and ApoG2, as indicated, and processed as described in the Materials and Methods. Values reported are ±SEM of three independent experiments.

Treatment of normal breast epithelial MCF10A cells with increasing concentrations of gossypol and ApoG2 shows that the compounds do not cause any significant inhibition of MCF10A cell growth (Figure 1a). However, both gossypol and ApoG2 cause a significant reduction in proliferation in pancreatic BxPC3, breast MDA-MB-231 and prostate PC3 cancer cell lines in a dose-dependent manner (Figure 1b,c), as assessed by the MTT assay. These results demonstrate a cancer-specific activity of these compounds. Furthermore, in order to compare the apoptosis-inducing potential of the two compounds in these cancer cell lines, Histone-DNA ELISA was performed. Apoptosis-induction in cells is a well-orchestrated event that is characterized by the condensation of membrane blebbing, cytoplasm condensation and activation of endonucleases. These endonucleases cleave the double-stranded DNA at the accessible points between nucleosomes, leading to the generation of DNA fragments in multiples of 180-bp subunits. Interestingly, the generation of these fragments in apoptotic cells begins several hours before plasma membrane breakdown. These histone-DNA complexes are then quantified based on the quantitative “sandwich enzyme immunoassay” principle using anti-histone-biotin and anti-DNA-peroxidase with ABTS as the substrate. As can be observed from the results given in Figure 2, treatment of the cancer cells with increasing concentrations of the compounds (0–20 μM) for 72 h led to a progressive increase in the absorbance at 405 nm, indicating increased apoptosis. ApoG2 was found to induce a significantly greater degree of apoptosis in all the cancer cell lines tested. As 5 μM concentration of gossypol and ApoG2 was found to induce more than 50% growth inhibition in most of the cancer cell lines tested, further studies with these compounds on cell lines were done at 5 μM concentration.

### 2.2. Neocuproine Inhibits Cell Proliferation and Apoptosis Induction by Gossypol/ApoG2 in Cancer Cell Lines

**Figure 3:**
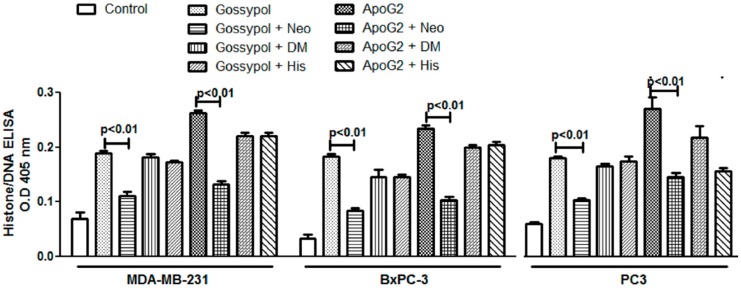
Cancer cell lines were incubated with 50 μM redox-metal-specific chelators, and treated with 5 μM gossypol/ApoG2 and further processed as described in the Materials and Methods. Neo: neocuproine; DM: desferrioxamine mesylate; His: Histidine. All results presented are mean (±SEM) of three independent experiments.

Since we have previously observed that copper chelation by neocuproine and bathocuproine reduced the extent of *in vitro* cellular DNA breakage by gossypol and ApoG2 in isolated peripheral lymphocytes [31], we were interested to investigate the effect of metal chelators on apoptosis induction by these compounds in cancer cell lines. In the experiment shown in Figure 3, it is seen that copper chelator neocuproine is able to protect the cancer cells against the cytotoxic action and apoptosis induction of both gossypol, as well as ApoG2, whereas iron chelator desferrioxamine mesylate and zinc chelator histidine do not provide any significant inhibition against gossypol/ApoG2 induced apoptosis in cancer cell lines; thus confirming that the anticancer activity of the parent compound gossypol and its derivative ApoG2 involves mobilization of endogenous copper.

### 2.3. Gossypol/ApoG2-Induced Cell Death Involves the Generation of Reactive Oxygen Species

Since copper levels are considerably elevated in various malignancies [32], cancer cells may be subject to greater electron transfer between copper ions and gossypol/ApoG2 to generate ROS. As summarized in Table 1, all the three scavengers of ROS, *i.e.*, thiourea (TU), superoxide dismutase (SOD), and catalase (Cat), inhibit gossypol/ApoG2-induced apoptotic activities in all three cancer cell lines. Our data indicates that ROS mediate the induction of apoptosis by gossypol and ApoG2 in these cancer cell lines. Superoxide anion generation spontaneously leads to the formation of H_2_O_2_, which, in turn, oxidizes reduced copper to generate hydroxyl radicals in a Fenton-like type reaction. Cancer cells have an imbalance in antioxidant enzymes compared with normal cells [33]. In cancer cells, enhanced ROS levels can overwhelm the cells’ antioxidant capacity, leading to irreversible damage and apoptosis [34].

### 2.4. Neocuproine Decreases Gossypol/ApoG2-Induced Suppression of Clonogenic Potential of Cancer Cell Lines

**Figure 4:**
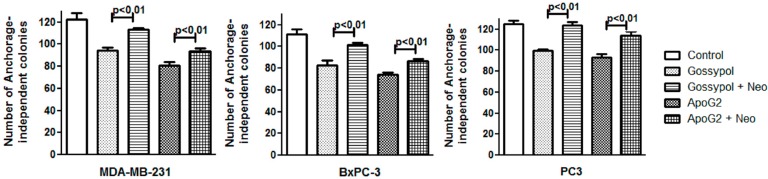
Cancer cell lines were plated in 24-well plates as described in the Methods. Culture was supplemented with 5 μM gossypol/ApoG2 with or without metal chelator neocuproine (Neo, 50 μM). Colonies (>50 cells), after appropriate culture time (22 days), were counted. Experiments were done in quadruplicate and mean values are reported. *p* < 0.01 when compared to untreated control cells.

*In vitro* tumorigenecity assay or anchorage-independent clonogenic assay is done to identify the ability of a single cell culture in a three-dimensional environment devoid of attachment to form a multicellular colony. The assay is a method of choice to determine the tumorigenic/reproductive ability of a cell during treatment with a cytotoxic agent. As shown in Figure 4, treatment of cancer cell lines MDA-MB-231, BxPC3 and PC3 with gossypol and ApoG2 resulted in a decrease of anchorage-dependent colonies. However, the treatment of cells with gossypol/ApoG2 in presence of neocuproine leads to a significant increase in the number of colonies; again suggesting the role of copper in gossypol and ApoG2 induced cell growth inhibition.

### 2.5. Copper Supplementation to ’Normal’ Breast Epithelial MCF10A Cells Sensitizes Them to Treatment with Gossypol, ApoG2

**Figure 5:**
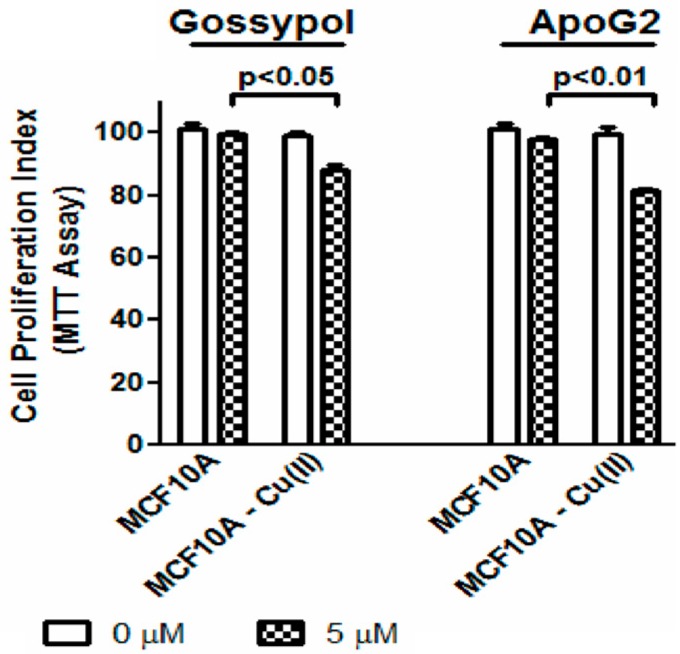
Normal breast epithelial MCF10A cells and MCF10A cells culture media supplemented with 25 μM of copper (designated as MCF10A-Cu) were treated with 5 μM gossypol/ApoG2 and cell growth inhibition assay was done by MTT assay as described in the Methods.

Normal breast epithelial MCF10A cells, which are known to have low levels of copper, were cultured in media supplemented with CuCl_2_ (copper supplemented cells are designated as MCF10A-Cu) and then treated with gossypol and ApoG2. Although copper itself is known to be cytotoxic at higher concentrations, Figure 5 indicates no significant difference in cell proliferation of MCF10A and MCF10A-Cu cells. However, when the MCF10A and MCF10A-Cu were treated with the test compounds, a significant inhibition in cell growth of MCF10A-Cu cells was seen; thus suggesting that supplementation of copper resulted in increased sensitivity and cytotoxicity of the normal MCF10A cells against these test agents. This further confirms the idea that the anticancer activity of these compounds is mediated by copper.

## 3. Discussion

We have previously observed that gossypol and ApoG2 are capable of inducing copper-mediated oxidative DNA breakage in isolated peripheral lymphocytes [31]. Based on our previous observations, we further investigated the effects and mechanism of action of these compounds in cancer cell lines. Herein, we observed that both gossypol and ApoG2 significantly inhibit growth and induce apoptosis in breast, pancreatic and prostate cancer cell lines. Moreover, the semi-synthetic derivative ApoG2 was observed to be more potent than gossypol in inhibiting cell growth. Apoptosis induction in cancer cells by gossypol and ApoG2 could be inhibited on incubation with copper-sequestering agent neocuproine, whereas iron and zinc chelators were relatively ineffective in any of the cell lines tested. Moreover, co-treatment of cells with ROS scavengers and gossypol/ApoG2 also reduced the induction of apoptosis in these cells. Thus, gossypol and ApoG2 function similar to plant polyphenolics, such as catechins, isoflavones, stilbenes, flavonoids, *etc.*, which can mobilize cellular endogenous copper generating ROS with consequent cell death [9,10,11,12,13,20,31,35,36,37]. This could indeed be an important mechanism of the anticancer property of antioxidants molecules.

Similar to previously published studies [38], we observed that gossypol and ApoG2 did not affect the proliferation of normal breast epithelial cells. This observation can, in part, be explained by the negligible levels of copper present in these cells as compared to breast cancer cells [39]. We had earlier proposed that the ability of polyphenolic antioxidants to specifically target cancer cells lies in the unique enrichment of copper in these cancer cells [9]. Thus, with the increase in copper concentrations in cancer cells, the cytotoxic concentration of polyphenolic antioxidants would be lower. Furthermore, we have previously demonstrated that copper supplementation to MCF10A cells enhanced the expression of the membrane-bound copper transporter Ctr1, potentially increasing copper uptake and accumulation in these cells, thereby, sensitizing cells to the cytotoxic action of polyphenols [9]. Similar to these observations, in this study, we also observed the sensitization of copper supplemented MCF10A (MCF10A-Cu) cells towards gossypol and ApoG2.

Pioneering studies by Folkman [40,41] established that copper is the simplest angiogenic molecule. Furthermore, growth factors identified as mediators of angiogenesis, ceruloplasmin, heparin, and tripeptide glycly-histidyl-lysine, are copper binding proteins and are known to be non-angiogenic when not bound with copper; they, however, become angiogenic when copper bound [42]. Copper metabolism and distribution have, indeed, been found to be altered in tumor bearing mice, rats and humans [43,44]. Moreover, the alteration in copper metabolism is currently being developed as a potential biomarker for molecular cancer imaging in humans [7]. While the concentration of zinc and selenium are significantly lower in cancer [45], the concentration of copper was almost always found to be significantly elevated (up to two- to three-fold) in tumor samples compared to age-matched normal tissue [32]. Furthermore, in order to target the angiogenic potential of copper and to ”starve” cancer, copper chelators, such as clioquinol, tetrathiomolybdate, *etc.*, have been demonstrated to reduce the growth of tumor cells *in vitro* and *in vivo* [46,47,48,49,50,51]. Apart from angiogenesis-promoting properties of copper, it has also been recognized to have a central role in intracellular signaling and tumor metastasis by participating in the transcriptional regulation of E-cadherin [52]. Apart from being one of the essential nutritional metals for the functions of numerous proteins, copper is also observed to be closely associated with guanine bases in DNA [53].

**Figure 6:**
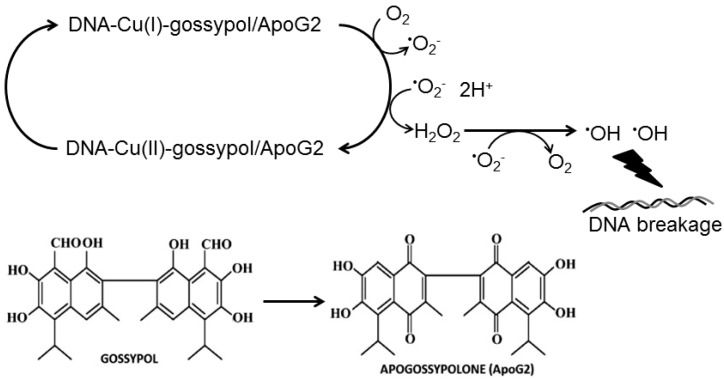
The redox cycling of the ternary complex involving gossypol/ApoG2, copper ions and DNA leading to the generation of various ROS species has been demonstrated in the schematic model along with the chemical structures of gossypol and ApoG2.

Cu^2+^, being one of the most redox-active metal ions in living cells, can be subjected to greater electron transfer under the influence of antioxidants in cancer cells to generate ROS. The very property of antioxidants that entails them to scavenge free radicals is the ease with which they can undergo metal-mediated generation of reactive species. The generation of site-specific hydroxyl radicals in a copper mediated Fenton reaction is capable of inducing apoptosis in thymocytes [54]. Moreover, thiol-containing compounds were observed to induce apoptosis in different cell lines only in the presence of free copper or ceruloplasmin, but not free iron or transferrin [55]. We have demonstrated significantly that the antioxidant-Cu(II) complex can induce redox cycling of copper by transient reduction of Cu(II) to Cu(I) with subsequent ROS generation. The generated ROS then subsequent act as effectors of antioxidant-induced DNA breakage and cell death in a mechanism independent of Fas and mitochondria mediated apoptosis. Our present results point to a similar generation of ROS and the resulting DNA damage by gossypol/ApoG2 (Figure 6). Studies have demonstrated that increase in intracellular ROS can induce apoptosis independent of caspases [56,57,58]. Furthermore, as ROS act as secondary messengers in the cellular systems, the modulation of genes (e.g., P53, NF-κB, c-MYC, *etc.*) and the apoptotic genes on polyphenol treatment may be caused by the secondary effects of ROS.

Thus, we can conclude that the major significance of the presented results lies in demonstrating that the antioxidants, gossypol and its derivative, ApoG2, are capable of mobilizing and redox-cycling the endogenous copper in cancer cells, causing the generation of ROS, leading to oxidative cell death. This study also provides the rationale for subsequent utilization of the molecules as parent structures for the synthesis of better and novel anticancer agents with better copper-chelating and ROS-generating properties and longer half-lives. Due to the pre-existing oxidative stress overload in tumor cells, any further increase in ROS levels can lead to cytotoxicity [59]. Thus, the mechanism of antioxidant mediated-mobilization and the reduction of endogenous copper proposed by us is a non-enzymatic and copper-mediated pathway for cytotoxic action of anticancer agents with antioxidant/pro-oxidant capacity.

## 4. Materials and Methods

### 4.1. Cell Lines and Reagents

Cancer cell lines MDA-MB-231 (breast cancer); BxPC-3 (pancreatic cancer); PC3 (prostate cancer) were obtained from ATCC (Manassas, VA, USA). MDA-MB-231 and BxPC-3 cells were maintained in DMEM (Invitrogen, Carlsbad, CA, USA); PC3 cells were maintained in RPMI 1640 (Invitrogen, Carlsbad, CA, USA). Both of these media were supplemented with 10% fetal bovine serum (FBS), 100 units/mL penicillin and 100 μg/mL streptomycin. The normal breast epithelial cell line, MCF10A, was maintained in DMEM/F12 (Invitrogen, Carlsbad, CA, USA), supplemented with 5% horse serum, 20 ng/mL EGF, 0.5 µg/mL hydrocortisone, 0.1 µg/mL cholera toxin, 10 µg/mL insulin, 100 units/mL penicillin, and 100 µg/mL streptomycin. All cells were cultured in a 5% CO_2_-humidified atmosphere at 37 °C. Stock solutions of gossypol and ApoG2 were always made fresh at a concentration of 25 mM in DMSO. Stock solutions of metal ion chelators (50 mM) were also freshly prepared for individual assays in PBS (phosphate-buffered saline).

### 4.2. MTT Assay

MDA-MB-231, BxPC-3 and PC3 cells were seeded overnight at a density of 2 × 10^3^ cells per well. After seeding, fresh culture media, containing either vehicle control DMSO or the test compounds, was added to wells. Metal ion chelators were added at indicated concentrations in individual assays. After 72 h of incubation, 25 μL MTT (3-(4,5-dimethylthiazol-2-yl)-2,5-diphenyltetrazolium bromide) solution (from a stock solution of 5 mg/mL in PBS) was added to individual wells and incubated at 37 °C for another 2 h. Thereafter, supernatant was aspirated and 100 μL of DMSO added to dissolve the MTT formazan by mixing on a rotating shaker for 30 min. Absorbance (595 nm) was measured on Multifunctional Microplate Reader (TECAN, Durham, NC, USA). Inhibition of cell growth in normal breast epithelial cells (MCF10A) and in MCF10A cells cultured in 25 µM CuCl_2_ (designated as MCF10A-Cu) by gossypol and ApoG2 was also done using an MTT assay, as described above.

### 4.3. Apoptosis Detection by Histone/DNA ELISA

Apoptosis was detected by Cell Death Detection ELISA Kit (Roche, Palo Alto, CA, USA). For this assay, cells were treated with either DMSO control or the test compounds for 72 h, followed by extraction of cytoplasmic histone/DNA fragments and their incubation in microtiter plate modules that were coated with anti-histone antibody. Immobilized histone/DNA fragments were detected by peroxidase-conjugated antibody and a color was developed with an ABTS (2,2′-azino-bis(3-ethylbenzothiazoline-6-sulphonic acid) substrate, which was read at 405 nm using a Multifunctional Microplate Reader (TECAN, Durham, NC, USA).

### 4.4. Soft Agar Colonization Assays

In individual wells of 24-well culture plates, 0.5 mL culture media, with 0.3% (*w*/*v*) agar and 3 × 10^4^ cells, were layered over a basal layer that contained 0.7% (*w*/*v*) agar with normal culture media. At the time of seeding, the culture was supplemented with 5 µM of the gossypol or ApoG2 or the vehicle DMSO, in the presence or absence of metal chelators. Colonies (>50 cells) were counted after an appropriate culture time (22 days). Culture media, with appropriate inhibitors, was replenished twice a week. Experiments were carried out in quadruplicate, and mean values are reported.

### 4.5. Statistical Analysis

Results are expressed as mean ± SEM of at least three independent observations. Student’s *t*-test was used to examine statistically significant differences. Analysis of variance was performed using ANOVA. *p*-values ≤ 0.05 were considered statistically significant.

## Figures and Tables

**Table 1:** Effect of reactive oxygen species (ROS) scavengers on gossypol and ApoG2-induced apoptosis in cancer cell lines. Cancer cell lines were incubated with different ROS scavengers, and treated with gossypol/ApoG2, as described in the Methods. Cell Death Detection ELISA Kit was used to assess the induction of apoptosis, as described above. Gossypol/ApoG2, 5 μM; TU, 700 μM thiourea; SOD, 100 μg/mL superoxide dismutase; Cat, 100 μg/mL catalase. *p* < 0.05 compared to treated control.

Cell Lines	Treatment	Apoptosis (Folds) ^#^	Effect of Scavengers (% Inhibition)
**MDA-MB-231**	**Untreated**	1.0	
	Gossypol	2.7	–
	+ TU	1.65	38.89
	+ SOD	1.62	40
	+ Cat	1.58	41.48
	ApoG2	3.4	54.41
	+ TU	1.55	55.29
	+ SOD	1.52	56.47
	+ Cat	1.48	–
**BxPC-3**	**Untreated**	1.0	
	Gossypol	2.1	–
	+ TU	1.14	45.71
	+ SOD	1.66	20.95
	+ Cat	1.49	29.05
	ApoG2	2.6	–
	+ TU	1.35	48.08
	+ SOD	1.63	37.31
	+ Cat	1.58	39.23
**PC3**	**Untreated**	1.0	
	Gossypol	2.3	–
	+ TU	1.13	50.87
	+ SOD	1.68	26.96
	+ Cat	1.54	33.04
	ApoG2	2.9	–
	+ TU	1.38	52.41
	+ SOD	1.78	38.62
	+ Cat	1.53	47.24

^#^ Fold-apoptosis was calculated relative to untreated control by direct comparison of O.D. values at 405 nm (O.D. value of treatment group/O.D. value of untreated control). Inhibitory effect of scavengers (% Inhibition) was determined by the formula—[(a − b)/a] × 100, where *a* = fold apoptosis by Gossypol/ApoG2 and *b* = fold apoptosis in presence of scavenger (TU/SOD/Cat).

## Abbreviations

ApoG2: Apogossypolone
DMSO: Dimethyl Sulfoxide
ELISA: Enzyme-linked Immunosorbent Assay
ROS: Reactive Oxygen Species
MTT: 3-(4,5-dimethylthiazol-2-yl)-2,5-diphenyltetrazolium bromide
ATBS: 2,2′-azino-bis(3-ethylbenzothiazoline-6-sulphonic acid
